# Synthesis of a New Polyanion Possessing Dense 1,2,3-Triazole Backbone

**DOI:** 10.3390/polym13101614

**Published:** 2021-05-17

**Authors:** Linlin Xu, Yuri Kamon, Akihito Hashidzume

**Affiliations:** Department of Macromolecular Science, Graduate School of Science, Osaka University, 1-1 Machikaneyama-cho, Toyonaka, Osaka 560-0043, Japan; xul18@chem.sci.osaka-u.ac.jp (L.X.); kamon@chem.sci.osaka-u.ac.jp (Y.K.)

**Keywords:** polyanion, dense 1,2,3-traizole polymer, copper(I)-catalyzed azide–alkyne cycloaddition polymerization, metal ion adsorption

## Abstract

Polyanions are an important class of water-soluble polymers because polyanions are utilized in a wide range of industrial fields. It is thus a great challenge to develop polyanions with novel structures to make their applications broader. In this study, a new polyanion with a dense 1,2,3-triazole backbone, poly(4-azido-5-hexanoic acid) (poly(AH)), was synthesized by copper(I)-catalyzed azide–alkyne cycloaddition (CuAAC) polymerization of *t*-butyl 4-azido-5-hexanoate followed by hydrolysis of the *t*-butyl ester groups. Turbidimetric and potentiometric titration data indicated that poly(AH) was well soluble in water under basic conditions (pH < 7) and a weaker polyanion (apparent p*K*_a_ = 5.4) than polyacrylic acid (apparent p*K*_a_ = 4.5). Adsorption tests exhibited that sodium salt of poly(AH) (poly(AH)Na) adsorbed most preferably Fe^3+^ among the four metal ions examined, i.e., Cu^2+^, Pb^2+^, Li^+^, and Fe^3+^. ^1^H spin-lattice relaxation time measurements indicated that Fe^3+^ ions were adsorbed favorably onto the 1,2,3-triazole residues.

## 1. Introduction

Polyanions are an important class of water soluble polymers showing various effects, e.g., thickening, gelling, dispersing, cohesive, adhesive, moisturizing, and metal-ion scavenging effects. On the basis of the effects, polyanions are thus utilized in a wide range of industrial fields including detergents, cosmetics, pharmaceuticals, foods, paints, textiles, civil engineering, and construction [1,2,3,4,5,6]. There are a number of polyanions, which are categorized into natural, semi-synthetic, and synthetic polymers. Polyanions are composed of the polymer backbone, anionic residues, and linkers between the backbone and anionic residues. Polyanions are used in appropriate applications depending on their properties based on the chemical structure. Therefore, it is a great challenge to develop polyanions with novel structures to make their applications broader.

1,2,3-Triazole is an aromatic five-membered ring consisting of two carbon and three nitrogen atoms with a large dipole [7,8], which acts as an interaction site through hydrogen bonding, coordination bonding, dipole–dipole interaction, and π–π stacking [8,9,10,11,12,13,14]. 1,2,3-Triazoles are readily formed from azide and alkyne functionalities through 1,3-dipolar cycloaddition [7,15,16,17]. In particular, it is known that 1,4-disubstituted 1,2,3-triazoles are formed efficiently and selectively in the presence of copper(I) compounds [18,19,20]. This reaction is known as copper(I)-catalyzed azide–alkyne cycloaddition (CuAAC). Because of its tolerance, CuAAC has been used in a variety of fields as the most important reaction in click chemistry [21,22]. CuAAC has been also utilized for synthesis of polymers possessing 1,2,3-triazole units [23,24,25,26]. We have been working on synthesis of polymers with the backbone composed of dense 1,2,3-triazole units by CuAAC polymerization of 3-azido-1-propyne (AP) and its derivatives [27,28,29,30,31]. Unfortunately, AP polymer was insoluble in all the solvents examined presumably because of strong dipole–dipole interaction of dense 1,2,3-triazole units.

Recently, *t*-butyl 4-azido-5-hexanoate (tBuAH), which we designed and synthesized, was polymerized by CuAAC to yield a dense 1,2,3-triazole polymer soluble in organic solvents, including polar and halogenated ones, indicating that the *t*-butyl (*t*-Bu) ester side chains improved the solubility [32]. Since *t*-Bu ester is often used as a protecting group for carboxylic acid residue, a new polyanion, poly(4-azido-5-hexanoic acid) (poly(AH)), is obtained by hydrolysis of *t*-Bu ester, and the properties of poly(AH) are investigated preliminarily in this study.

## 2. Materials and Methods

### 2.1. Materials

Tetrahydrofuran (THF), sodium (+)-ascorbate (NaAsc), ethylenediaminetetraacetic acid (EDTA), *N*,*N*-dimethylformamide (DMF), and dichloromethane (DCM) were purchased from FUJIFILM Wako Pure Chemical Corp. (Osaka, Japan). Trifluoroacetic acid (TFA), copper(I) bromide (CuBr), and lithium chloride (LiCl) were purchased from Nacalai Tesque Co., Ltd. (Kyoto, Japan). Lead(II) chloride (PbCl_2_) and iron(III) nitrate (Fe(NO_3_)_3_·9H_2_O) were purchased from Sigma-Aldrich (St. Louis, MO, USA). Water was purified with a Millipore Milli-Q system. Other reagents were used as received.

*t*-Butyl 4-azido-5-hexynoate (tBuAH) was prepared according to the procedure reported previously [32].

### 2.2. Measurements

^1^H NMR spectra were measured on a JEOL JNM ECA400 spectrometer (JEOL Ltd., Tokyo, Japan) using tetramethylsilane (TMS) as an internal standard in CDCl_3_ as a solvent at 25 °C. Pulse-field-gradient spin-echo (PGSE) NMR data were obtained on a JEOL JNM ECA500 spectrometer (JEOL, Ltd., Tokyo, Japan) at 25 °C using deuterium oxide (D_2_O) as a solvent. The bipolar pulse pair stimulated echo (BPPSTE) sequence was applied [33,34,35]. The strength of pulsed gradients (*g*) was increased from 0.3 to 90 gauss cm^–1^. The time separation of pulsed field gradients (Δ) and their duration (*δ*) were 0.15 and 0.002 s, respectively. The sample was not spun and the airflow was disconnected. The shape of the gradient pulse was rectangular, and its strength was varied automatically during the course of the experiments. Two-dimensional diffusion ordered spectroscopy (2D DOSY) data were obtained by inverse Laplace transformation using the SPLMOD method [36,37]. ^1^H spin-lattice relaxation times (*T*_1_) were determined using a conventional inversion recovery technique with a 180°-*τ*-90° pulse sequence [38,39,40]. Size exclusion chromatography (SEC) analysis was carried out at 25 °C on a TOSOH HLC-8320GPC system equipped with an RI detector and TOSOH TSK SuperAWM-H columns (TOSOH Corp. (Tokyo, Japan)), using dimethyl sulfoxide (DMSO) containing lithium bromide (1.05 g L^–1^) as the eluent at a flow rate of 0.4 mL min^–1^. The molecular weights were calibrated with seven standard samples of poly(ethylene glycol) (PEG) and poly(ethylene oxide) (PEO) (Scientific Polymer Products, Inc., Ontario, NY, USA)). The *M*_w_ values for standard samples were 9.6 × 10^2^, 1.45 × 10^3^, 3.07 × 10^3^, 7.29 × 10^3^, 1.19 × 10^4^, 2.12 × 10^4^, and 1.132 × 10^5^. Sample solutions were filtrated with a DISMIC-13JP PTFE 0.50 μm filter just prior to injection. Fourier transform infrared (FTIR) spectra were recorded on a JASCO FT/IR-4600 spectrometer (JASCO Corp., Tokyo, Japan) equipped with a JASCO ATR PRO ONE cell.

### 2.3. Preparation of Poly(4-azido-5-hexynoic acid) (Poly(AH))

**Scheme 1:**
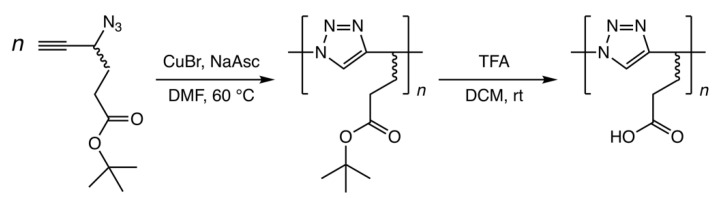
Synthesis of poly(AH) by CuAAC polymerization of tBuAH followed by hydrolysis of the *t*-Bu ester.

A typical procedure for preparation of poly(AH) is described below (Scheme 1).

CuBr (39 mg, 10% eq) and NaAsc (160 mg, 30% eq) were added to a solution of tBuAH (560 mg, 2.7 mmol) in DMF (0.2 mL) under a nitrogen atmosphere. The reaction mixture was warmed using an oil bath thermostated at 60 °C with stirring for 48 h. After the reaction mixture was cooled down to room temperature, ethyl acetate (20 mL) was added to the mixture. The organic layer was washed with 0.50 M EDTA (2 × 10 mL) and with water (100 mL). The organic phase was dried with Na_2_SO_4_. The polymer obtained was recovered by reprecipitation with hexane (60 mL). The polymer was dried at 40 °C under reduced pressure (360 mg, 64%). ^1^H NMR (CDCl_3_, 400 MHz) *δ* (ppm): 7.94 (1H, 1,2,3-triazole CH), 5.99 (1H, CH), 2.57 (2H, CH_2_), 2.17 (2H, CH_2_), 1.41 (9H, *t*-C_4_H_9_).

The poly(tBuAH) sample (155 mg, 0.74 mmol monomer units) was dissolved in DCM (3.7 mL). TFA (3.7 mL) was added to the solution. The reaction mixture was stirred for 60 min. The reaction mixture was diluted with methanol (100 mL) and the solvent was removed under reduced pressure to obtain the product, poly(AH). Poly(AH) was recovered as colorless solid after drying at 60 °C under reduced pressure (112.6 mg, 99% yield). ^1^H NMR (D_2_O, 400 MHz) *δ* (ppm): 8.35 (1H, 1,2,3-triazole CH), 6.06 (1H, CH), 2.62 (2H, CH_2_), 2.10 (2H, CH_2_).

The poly(AH) sample was then neutralized with an equimolar amount of 0.10 M NaOH, and the poly(AH) neutralized (poly(AH)Na) was then recovered by drying under reduced pressure at 45 °C for 12 h.

### 2.4. Turbidimetric and Potentiometric Titrations

A sample of poly(AH) not neutralized (68 μmol monomer units) was dissolved in 0.10 M NaOH (780 μL) and then diluted with water (5.4 mL). While the polymer solution was titrated with 0.10 M HCl from pH 10.9 using a microburet at 25 °C under a nitrogen atmosphere, turbidity and pH were monitored after establishing the equilibrated state, in which the pH reached a constant value after each step of titrant addition. Turbidities, reported as 100–%*T*, were measured with a Brinkmann PC920 probe colorimeter equipped with a 1 cm path length fiber optics probe at 620 nm. Values of pH were measured with a Horiba F-23 pH meter equipped with a Horiba 9618S-10D glass electrode. The reference electrode was calibrated with buffer solutions of pH 4.01, 6.86, and 9.18 prior to pH measurements. The degrees of neutralization (α) were calculated from the amounts of the monomer unit and the added HCl.

### 2.5. Adsorption Tests

Poly(AH)Na (11.5 mg, 65.7 μmol monomer units) was added to an aqueous solution of a salt (100 mg L^–1^ metal ion), i.e., PbCl_2_, CuSO_4_·5H_2_O, (FeNO_3_)_3_·9H_2_O, or LiCl, at pH = 7. The mixture was stirred at 35 °C for 12 h. After reaching equilibrium, 4 mL of the mixture solution was taken. The polymer–metal ion complexes were removed with a Pall Microsep^TM^ advanced centrifugal device 1K Omega (1 kDa cut off) by centrifugation at 1000 rpm for 15 min. The filtrate obtained (1.2 mL) was analyzed by a PerkinElmer Optima 8300 ICP-Optical Emission Spectroscopy system (PerkinElmer Inc. (Waltham, MA, USA)) to evaluate the equilibrium concentration (*c*_e_) of metal ion. The concentrations of metal ions in the equilibrated state were reported as average values of three measurements. The removal ratio (*r*_removal_) and adsorption capacity (*q*_e_) were calculated by

(1) $$r_{\mathrm{removal}}=\frac{100\left(c_{i}-c_{e}\right)}{c_{i}}$$

(2) $$q_{e}=\frac{V\left(c_{i}-c_{e}\right)}{m}$$

where *c*_i_ is the initial ion concentration, *m* is the weight of poly(AH), and *V* is the volume of solution.

## 3. Results and Discussion

### 3.1. Synthesis and Structural Characterization of Poly(AH)

In our previous study, we synthesized tBuAH possessing *t*-butyl (*t*-Bu) ester in the side chain and conducted its CuAAC polymerization to obtain poly(tBuAH) with a dense 1,4-disubstituted 1,2,3-triazole backbone [32]. The unsubstituted dense 1,4-disubstituted 1,2,3-triazole polymer (poly(3-azido-1-propyne)) was insoluble in all the solvents studied, whereas poly(tBuAH) was soluble in many organic solvents, including polar and halogenated solvents, presumably because of the *t*-Bu ester moiety in the side chain. In this study, a new polyanion, poly(AH), was synthesized by hydrolysis of the *t*-Bu ester moieties in poly(tBuAH). According to the procedure reported previously [32], tBuAH was polymerized by CuAAC to yield poly(tBuAH) consisting of 1,4-disubstituted 1,2,3-triazole residues. The *M*_w_ values for the poly(tBuAH) samples obtained were determined to be 7.9 × 10^3^ and 8.8 × 10^3^, respectively, by SEC (Table 1). The poly(tBuAH) samples were dissolved in DCM, and the *t*-Bu ester groups were hydrolyzed with TFA to synthesize poly(AH) samples. Since poly(AH) is a weak polyanion possessing carboxylic acid moieties, the poly(AH) samples were neutralized with an equimolar amount of NaOH to dissolve in water and then recovered as a salt-type polymer samples (poly(AH)Na) by drying under reduced pressure.

**Figure 1:**
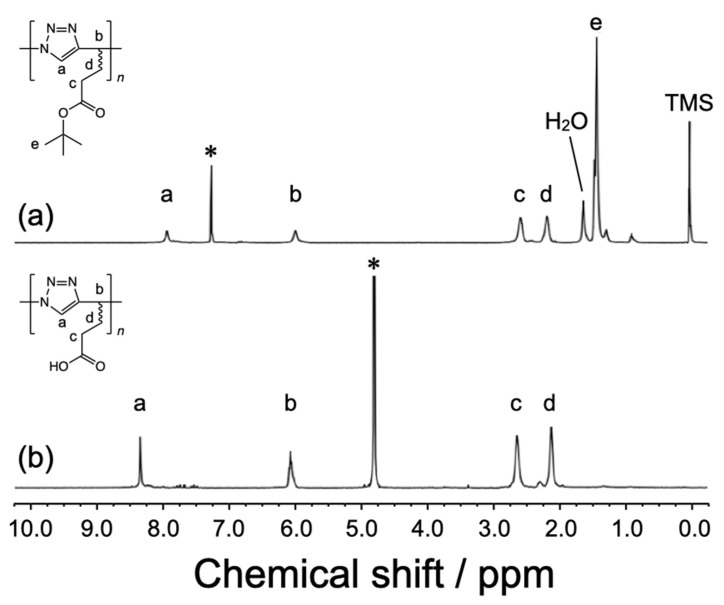
^1^H NMR spectrum for poly(tBuAH) (CDCl_3_) (**a**) and poly(AH) (D_2_O) (**b**). Assignments are indicated as a, b, c, d, and e. Asterisks denote the signals due to the residual protons in solvents.

Poly(AH) was characterized by ^1^H NMR and FTIR. Figure 1 shows ^1^H NMR spectra for poly(tBuAH) and poly(AH). As can be seen in Figure 1a, the spectrum of poly(tBuAH) in CDCl_3_ contains signals ascribable to the proton in 1,4-disubstituted 1,2,3-triazole and the methine proton in the main chain at ca. 7.9 and 6.0 ppm, respectively. The signals at ca. 2.6 and 2.2 ppm are assignable to the protons of two methylenes in side chain. The signal due to the *t*-Bu ester was observed at ca. 1.4 ppm. This ^1^H NMR spectrum is thus indicative of successful preparation of poly(tBuAH) consisting of 1,4-disubstituted 1,2,3-triazole units. In the spectrum of poly(AH) in D_2_O (Figure 1b), on the other hand, signals due to protons of the triazole, methine, and methylenes were observed, whereas the signal ascribable to the *t*-Bu ester was not observed. These observations indicate that poly(AH) was successfully obtained through quantitative hydrolysis of the *t*-Bu ester groups, as can be seen in Scheme 1.

**Figure 2:**
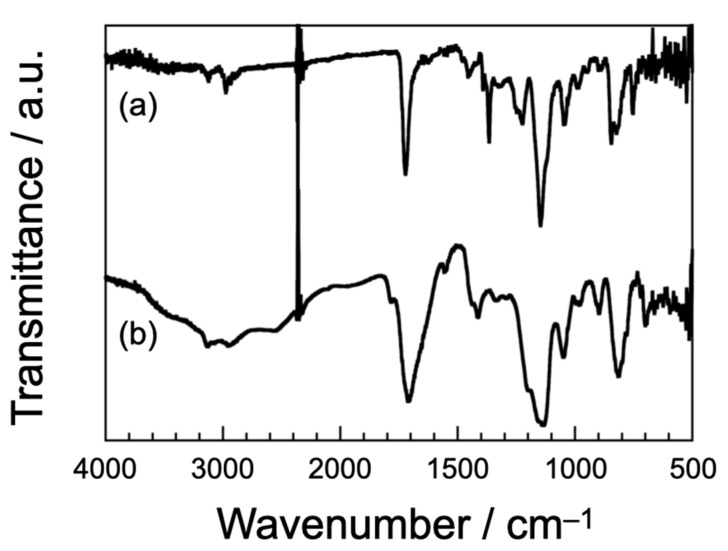
FTIR spectrum for poly(tBuAH) (**a**) and poly(AH) (**b**).

Figure 2 shows FTIR spectra of poly(tBuAH) and poly(AH). Both the spectra indicate absorption bands ascribable to 1,2,3-triazole at ca. 1500, 1000, and 800 cm^–1^. In the spectrum of poly(tBuAH), the signal attributed to the stretching vibration of the ester carbonyl was observed around 1720 cm^–1^, whereas in the spectrum of poly(AH), a broad signal attributed to the carboxylic acid was observed around 1692 cm^–1^. It should be noted here that the spectrum of poly(AH) contains a broad absorption band ascribable to the stretching vibration of O–H in the region of 2500–3500 cm^–1^ caused by intermolecular hydrogen bonding. These FTIR spectra also confirm that poly(AH) was successfully obtained by quantitative hydrolysis of the *t*-Bu ester groups in the side chain of poly(tBuAH) while the dense 1,2,3-triazole backbone was maintained.

### 3.2. Turbidimetric and Potentiometric Titrations

**Figure 3:**
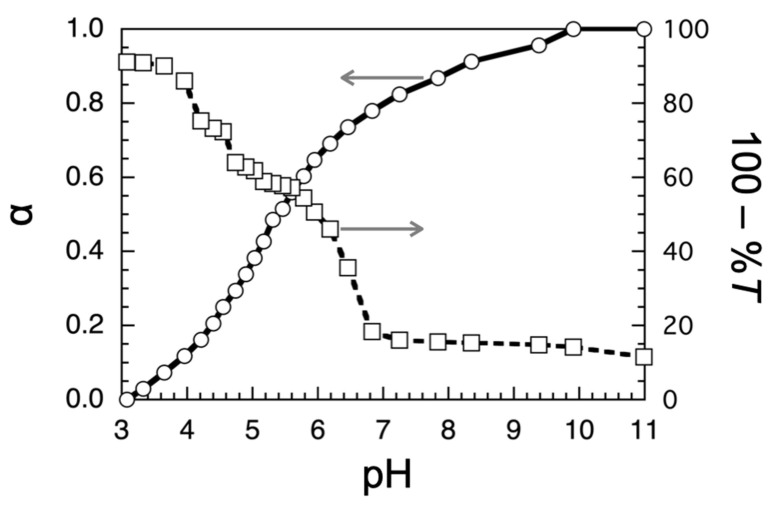
Turbidimetric and potentiometric titration data for an aqueous solution of poly(AH). The titration was performed by stepwise addition of 30 μL of 0.10 M HCl starting from pH 9.9.

Turbidimetric and potentiometric titrations were carried out to study basic characteristics of poly(AH) as polyanion in aqueous media. The acid form of poly(AH) (10.4 mg, 68 μmol monomer units) and a small excess of NaOH (0.10 M, 780 μL) were dissolved in water (5.4 mL). Values of turbidity and pH were recorded while hydrochloric acid (0.10 M) was added with a microburet to the polymer aqueous solution as a titrant with stirring under a nitrogen atmosphere. The dissociation degrees (*α*) were calculated using the mass of poly(AH) and the amount of HCl added, and the values of turbidity and *α* were plotted in Figure 3 against pH. As can be seen in Figure 3, the turbidity was almost 10% at pH > 7, whereas the turbidity increased with decreasing pH from 7 to 4 and then saturated at ca. 90% in the regime of pH < 4. On the other hand, *α* decreased with decreasing pH and reached 0 at ca. pH = 3. The apparent p*K*_a_ at which *α* = 0.5 was estimated to be ca. 5.4, which is larger than that of polyacrylic acid (PAA) (p*K*_a_ = 4.5) [41]. This observation indicates that poly(AH) is a weaker polyanion than PAA presumably because of the more hydrophobic backbone and linker of poly(AH) [42].

### 3.3. Pulse-Field-Gradient Spin-Echo NMR

**Figure 4:**
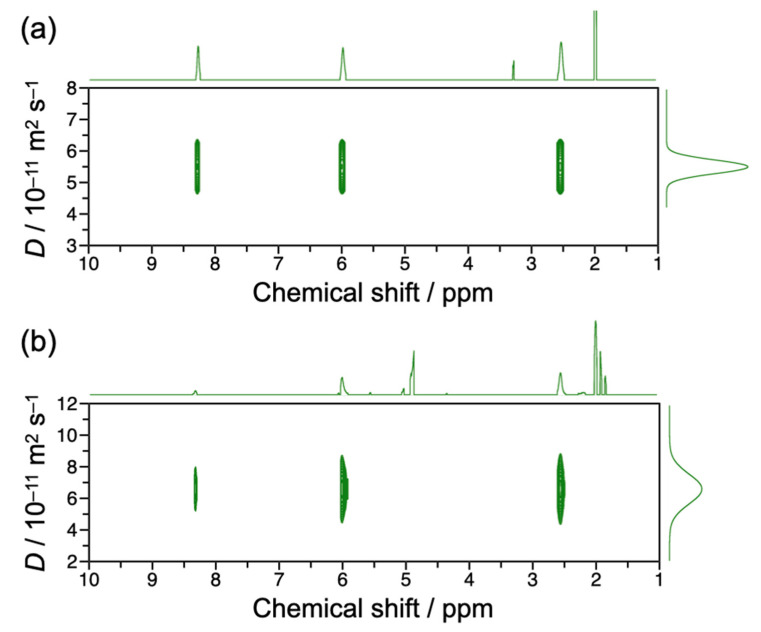
2D DOSY data for poly(AH)Na in D_2_O containing 5 mM NaCl at pH 9.0 (**a**) and 12.0 (**b**).

The molecular sizes of poly(AH) chains in aqueous solution at pH 9.0 and 12.0 (i.e., pD 9.4 and 12.4, respectively) were estimated by PGSE NMR using D_2_O containing 5 mM NaCl as a solvent. As can be seen in Figure 4, 2D DOSY data indicate that all the signals for poly(AH) were observed as unimodal distribution in the mutual diffusion coefficient (*D*) region of (4.5 − 6.5) × 10^−11^ and (4 − 9) × 10^−11^ m^2^ s^−1^ at pH 9.0 and 12.0, respectively. (Here, the signals for methylene protons at ca. 2.2 ppm were not observed because the signals were very close to that for acetonitrile, the internal standard.) Using the signal attributed to the main-chain methine proton, the *D* values were evaluated to be 5.2 × 10^−11^ and 4.7 × 10^−11^ m^2^ s^−1^ at pH 9.0 and 12.0, respectively. On the basis of these *D* values, the hydrodynamic radii values were calculated to be ca. 3.4 and 3.7 nm at pH 9.0 and 12.0, respectively, using the Einstein–Stokes equation. These data indicate that poly(AH) takes a conformation independent of pH under basic conditions.

### 3.4. Adsorption Tests

**Figure 5:**
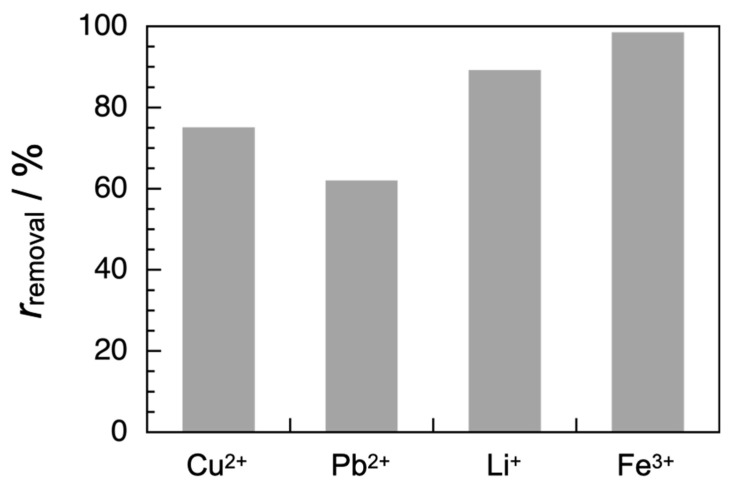
Removal ratio of poly(AH)Na for Cu^2+^, Pb^2+^, Li^+^, and Fe^3+^.

Poly(AH)Na possesses 1,2,3-triazole and carboxylate moieties in the monomer unit. Since 1,2,3-triazole and carboxylate moieties may act as relatively soft and hard ligands, respectively, poly(AH)Na may coordinate metal ions. Adsorption tests were thus performed using poly(AH)Na. Four salts, i.e., CuSO_4_·5H_2_O, PbCl_2_, LiCl, and Fe(NO_3_)_3_, were added to an aqueous solution of poly(AH)Na, stirred for 12 h to reach adsorption equilibrium, and then the polymer was removed by centrifugal filtration. The equilibrium concentrations (*c*_e_) of metal ions were determined by ICP measurements. Using the *c*_e_ value of metal ion, the removal ratio (*r*_removal_) and the amount of metal ion adsorbed on 1.0 g of poly(AH)Na (adsorption capacity, *q*_e_) were evaluated, as summarized in Table 2. The *q*_e_ values were 75.12 ± 0.02, 62.0 ± 0.2, 89.22 ± 0.02, and 98.57 ± 0.03 mg g^−1^ for the four metal ions used, i.e., Cu^2+^, Pb^2+^, Li^+^, and Fe^3+^, respectively (Figure 5). (Under the conditions in this study, the *r*_removal_ values were almost the same as the *q*_e_ values; 75.10 ± 0.02, 62.0 ± 0.2, 89.20 ± 0.02, and 98.54 ± 0.03% for Cu^2+^, Pb^2+^, Li^+^, and Fe^3+^, respectively.) These observations indicate that poly(AH)Na can be used as an adsorbent for metal ions, especially Fe^3+^ ion.

**Figure 6:**
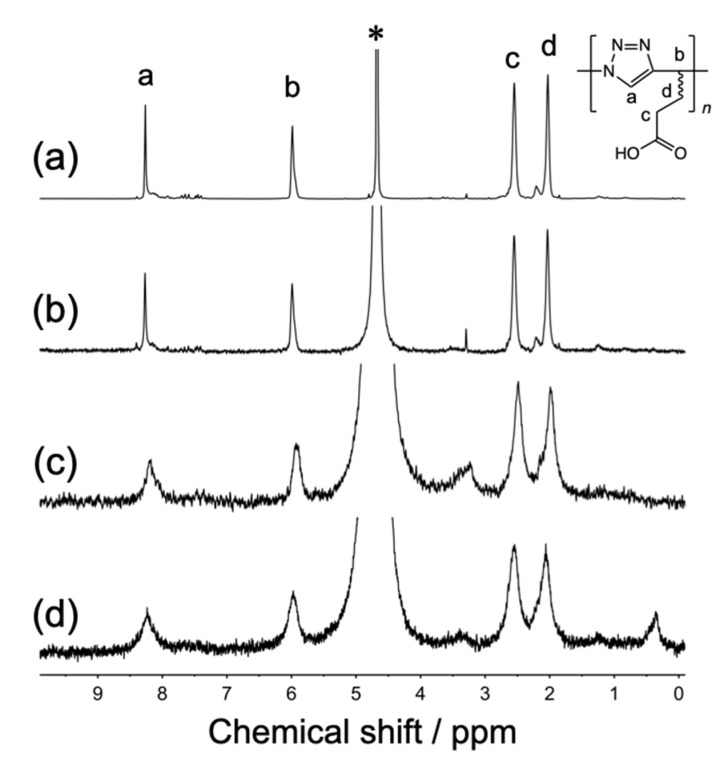
^1^H NMR spectrum for poly(AH)Na in the absence (**a**) and presence of 0.1 (**b**), 3.1 (**c**), and 9.1 mM of Fe(NO_3_)_3_ (D_2_O) (**d**). Assignments are indicated as a, b, c, and d. Asterisks denote the signals due to the residual protons in solvents.

**Figure 7:**
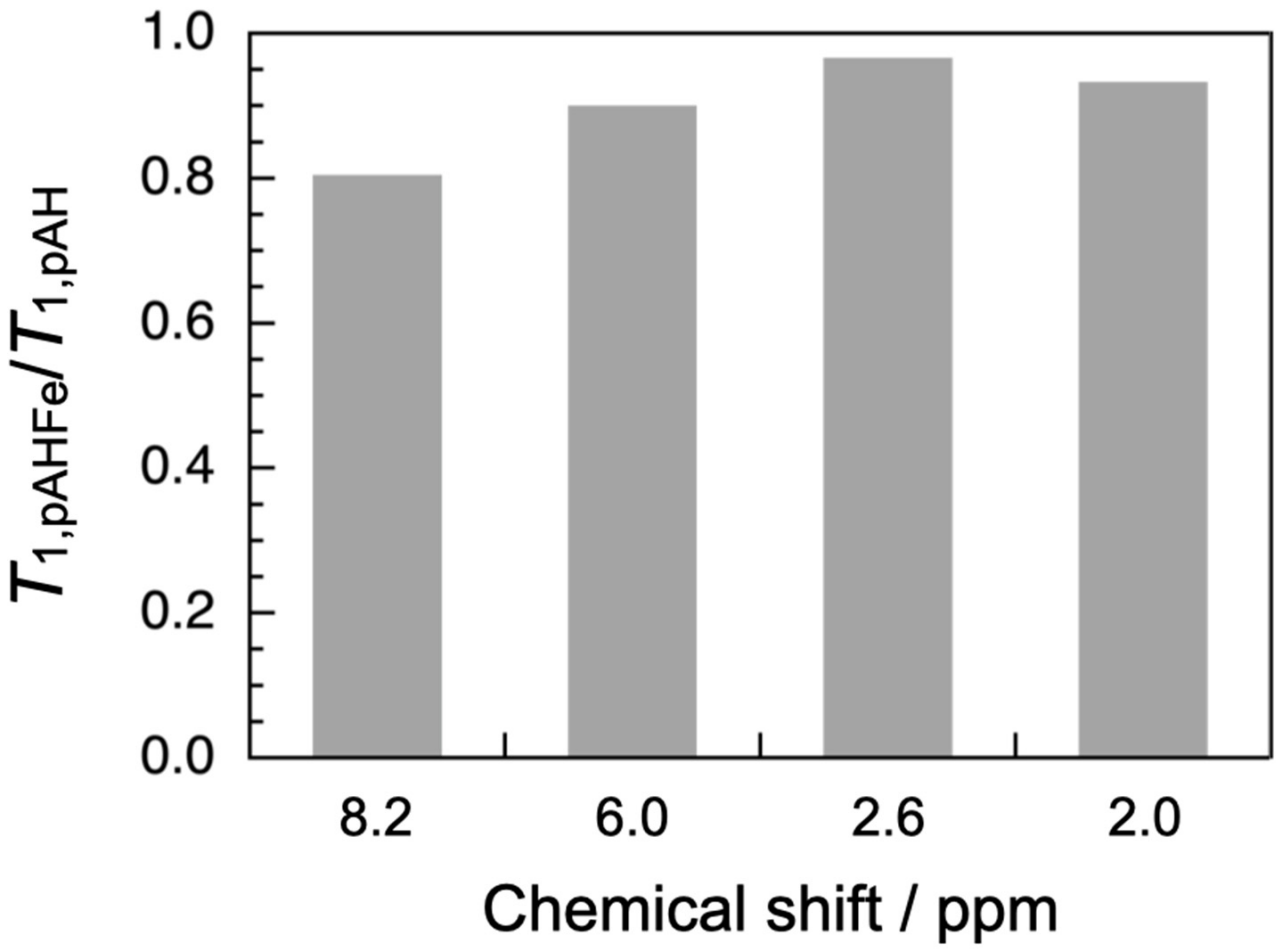
*T*_1,pAHFe_/*T*_1,pAH_ for ^1^H NMR signals.

Since Fe^3+^ is a paramagnetic species, NMR relaxation may be significantly faster in the presence of Fe^3+^. Figure 6 displays ^1^H NMR spectra for poly(AH)Na in the presence of varying concentrations of Fe(NO_3_)_3_. The signals due to poly(AH)Na are only slightly broader at [Fe^3+^] = 0.1 mM. At [Fe^3+^] = 3.1 and 9.1 mM, on the other hand, the ^1^H NMR spectra show markedly broader signals. It should be noted here that the signals due to the triazole and methine protons at ca. 8.2 and 6.0 ppm became weaker than did the signals due to the methylene protons at ca. 2.2. and 2.6 ppm. *T*_1_ values of signals were measured and compared in ^1^H NMR spectra in the presence and absence of 0.6 mM Fe^3+^ (Table 3). Figure 7 indicates the ratios of *T*_1_ values for the ^1^H NMR signals in the presence and absence of 0.6 mM Fe^3+^. The ratio was 80.4% for the triazole proton at ca. 8.2 ppm, whereas those were 90.0–96.6% for the methine (6.0 ppm) and methylene protons (2.6 and 2.0 ppm). These observations indicate that Fe^3+^ ions are adsorbed more preferably onto the 1,2,3-triazole residues [43,44].

## 4. Conclusions

As a new polyanion with a dense 1,2,3-triazole backbone, poly(AH), was synthesized by CuAAC polymerization of tBuAH followed by hydrolysis of the *t*-Bu ester groups. The ^1^H NMR and FTIR data confirmed the structure of poly(AH). Turbidimetric and potentiometric titration date indicated that poly(AH) was well soluble at pH > 7, slightly soluble in the pH region of 7–4, and practically insoluble in the regime of pH < 4. The apparent p*K*_a_ at which *α* = 0.5 was estimated to be ca. 5.4, which is larger than that of PAA (p*K*_a_ = 4.5), indicating that poly(AH) is a weaker polyanion than PAA presumably because of the more hydrophobic backbone and linker of poly(AH). On the basis of PGSE NMR data, the hydrodynamic radii of poly(AH)Na were estimated to be ca. 3.4 and 3.7 nm at pH 9.0 and 12.0, respectively, indicating that that poly(AH) takes a conformation independent of pH under basic conditions. Since 1,2,3-triazole and carboxylate moieties may act as relatively soft and hard ligands, respectively, adsorption tests were thus performed using poly(AH)Na. The amounts adsorbed were 75.12 ± 0.02, 62.0 ± 0.2, 89.22 ± 0.02, and 98.57 ± 0.03 mg g^−1^ for Cu^2+^, Pb^2+^, Li^+^, and Fe^3+^, respectively. These observations indicate that poly(AH)Na can be used as an adsorbent for metal ions, especially Fe^3+^ ion. The ratio of *T*_1_ values for the ^1^H NMR signals in the presence and absence of 0.6 mM Fe^3+^ was calculated to be 80.4% for the triazole proton at ca. 8.2 ppm, whereas those were 90.0–96.6% for the methine (6.0 ppm) and methylene protons (2.6 and 2.0 ppm), indicating that Fe^3+^ ions are adsorbed more preferably onto the 1,2,3-triazole residues. On the basis of poly(AH), synthesis of sequence-controlled amphiphilic polymers is in progress.

## Figures and Tables

**Table 1:** Basic characteristics of poly(tBuAH) samples prepared in this study.

Sample	*M*_w_/10^3 1^	*M*_w_/*M*_n_ ^1^
poly(tBuAH) ^2^	7.9	1.5
poly(tBuAH) ^3^	8.8	1.4

^1^ Determined by SEC measurements at 25 °C using DMSO containing 1.05 g L^–1^ LiBr as eluent. Molecular weights were calibrated with PEG and PEO standard samples. ^2^ The poly(AH) sample prepared from this poly(tBuAH) sample was used for the FTIR, turbidimetric and potentiometric titrations, and PGSE NMR. ^3^ The poly(AH) sample prepared from this poly(tBuAH) sample was used for the ^1^H NMR, adsorption tests, and *T*_1_ measurements.

**Table 2:** Removal percentages (*r*_removal_) and adsorption capacities (*q*_e_) of poly(AH)Na for metal ions.

Metal Ion	*c*_i_/mg L^−1^	*c*_e_/mg L^−1^	*r*_removal_/%	*q*_e_/mg g^−1^
Cu^2+^	100	24.90 ± 0.02	75.10 ± 0.02	75.12 ± 0.02
Pb^2+^	100	38.0 ± 0.2	62.0 ± 0.2	62.0 ± 0.2
Li^+^	100	10.80 ± 0.02	89.20 ± 0.02	89.22 ± 0.02
Fe^3+^	100	1.46 ± 0.03	98.54 ± 0.03	98.57 ± 0.03

**Table 3:** ^1^H NMR chemical shifts and *T*_1_ values for poly(AH)Na in the absence and presence of 0.6 mM Fe(NO_3_)_3_.

*δ*/ppm	*T*_1,pAH_ ^1^/s	*T*_1,pAHFe_ ^2^/s	*T*_1,pAHFe_/*T*_1,pAH_
8.2	1.47	1.18	0.804
6.0	1.22	1.10	0.900
2.6	0.376	0.363	0.966
2.0	0.485	0.452	0.932

^1^ *T*_1_ value for poly(AH)Na in the absence of Fe(NO_3_)_3_. ^2^ *T*_1_ value for poly(AH)Na in the presence of 0.6 mM Fe(NO_3_)_3_.

## Data Availability

Not applicable.
